# Cluster-specific genetic associations of *CDKAL1*, *CDKN2A*, *CDKN2B*, *HHEX*, *KCNQ1*, *MTNR1B*, *PAX4*, *SLC30A8*, *TCF7L2*, and *UBE2E2* variants in new onset type 2 diabetes

**DOI:** 10.1038/s41598-025-32840-y

**Published:** 2025-12-20

**Authors:** Nattachet Plengvidhya, Nipaporn Teerawattanapong, Tassanee Narkdontr, Saranya Innang, Suavaluk Songlilitchuwong, Sarocha Suthon, Watip Tangjittipokin

**Affiliations:** 1 Division of Endocrinology and Metabolism, Department of Medicine, Faculty of Medicine Siriraj Hospital, Mahidol University, Bangkok, Thailand; 2 Siriraj Center of Research Excellence for Diabetes and Obesity (SiCORE-DO), Research Department, Faculty of Medicine Siriraj Hospital, Mahidol University, Bangkok, Thailand; 3 Department of Research, Faculty of Medicine Siriraj Hospital, Mahidol University, Bangkok, Thailand; 4 Department of Immunology, Faculty of Medicine Siriraj Hospital, Mahidol University, Bangkok, 10700 Thailand

**Keywords:** Type 2 diabetes, Genetic association, SNP, Clusters, Precision medicine, Axiom array, Diseases, Endocrinology, Genetics

## Abstract

Type 2 diabetes (T2D) is a heterogeneous metabolic disorder. Recent cluster-based classifications offer insights into distinct pathophysiological subtypes. The objective of the study is to investigate the association of genetic variants in T2D-related genes with defined T2D clusters. We analyzed 678 single nucleotide polymorphisms (SNPs) from ten genes (*CDKAL1*, *CDKN2A*, *CDKN2B*, *HHEX*, *KCNQ1*, *MTNR1B*, *PAX4*, *SLC30A8*, *TCF7L2*, and *UBE2E2*) in 471 T2D patients classified into four clusters: Severe Insulin-Deficient Diabetes (SIDD), Mild Obesity-related Diabetes (MOD), Mild Age-related Diabetes (MARD), and Metabolic Syndrome-related Diabetes (MSD). Genotyping was performed using the Axiom PDMRAv2 array. Following Hardy–Weinberg Equilibrium filtering, 376 SNPs were analysed. The association between T2D clusters and SNPs was assessed by multinomial logistic regression. Nineteen SNPs showed significant differences in genotypic frequencies among clusters (*p* < 0.05). Eight SNPs (rs61875103 in *TCF7L2*; rs12576156, rs2283220, rs2074197, and rs163165 *KCNQ1*; rs4710943, rs9368248, and rs6456379 in *CDKAL1*) significantly associated with cluster assignment. Cluster-specific effects were most notable in SIDD and MOD subgroups. Our findings support genetic heterogeneity of *TCF7L2*, *KCNQ1*, and *CDKAL1* in T2D clusters and underscore the potential for genetically informed precision therapy strategies.

## Introduction

Type 2 diabetes (T2D) is a complex and multifactorial metabolic disorder characterised by insulin resistance, beta-cell dysfunction, or a combination of both^1^. While lifestyle and environmental factors play substantial roles in disease development, genetic predisposition significantly contributes to individual susceptibility and variation in clinical presentation^2^.

Traditional classification of T2D does not adequately capture the disease’s heterogeneity, often leading to generalised treatment strategies that may not suit all patients^3^. Recent efforts have introduced a cluster-based classification system that divides T2D into clinically distinct subgroups—such as Severe Insulin-Deficient Diabetes (SIDD), Mild Obesity-Related Diabetes (MOD), Mild Age-Related Diabetes (MARD), and Metabolic Syndrome-Related Diabetes (MSD) based on pathophysiological parameters including age at onset, BMI, HbA1c, insulin resistance, and beta-cell function^4^. This framework has been proposed to better understand disease mechanisms.

Despite growing interest in this cluster-based approach, the genetic architecture underlying these subtypes remains insufficiently explored, particularly in Southeast Asian populations. Most genetic studies of T2D have been conducted in European cohorts, raising concerns about the translatability of their findings to other ethnic groups^5^. The Thai population has distinct genetic backgrounds and lifestyle factors that may influence the expression and impact of T2D-associated genes^6,7^. Therefore, understanding genetic contributions to T2D subtypes in this population is critical for advancing precision medicine and ensuring equitable healthcare strategies^8^.

In this study, we focused on ten well-established T2D-associated genes—*CDKAL1*, *CDKN2A*, *CDKN2B*, *HHEX*, *KCNQ1*, *MTNR1B*, *PAX4*, *SLC30A8*, *TCF7L2*, and *UBE2E2*—that have been repeatedly implicated in genome-wide association studies (GWAS) across diverse populations^9–11^. These genes are known to play roles in insulin secretion (e.g., *TCF7L2*, *CDKAL1*, *KCNQ1*), beta-cell development (*HHEX*, *PAX4*), circadian rhythm (*MTNR1B*), and cell cycle regulation (*CDKN2A*, *CDKN2B*)^12^. We hypothesised that variants within these loci may exhibit differential associations across T2D clusters, potentially reflecting distinct biological pathways driving disease in each subgroup. By focusing on a Thai population, this study aimed to uncover population-specific genetic markers that could inform targeted therapeutic strategies and contribute to a more globally representative understanding of T2D pathogenesis.

## Materials and methods

### Study population

A total of 471 Thai individuals diagnosed with T2D were enrolled and categorised into four clusters (SIDD, MSD, MOD, and MARD) based on clinical parameters, including age at onset, BMI, HbA1c, insulin resistance, and beta-cell function. Type 2 diabetes was diagnosed according to the American Diabetes Association (ADA) guidelines^13^. The entire study was approved by the Siriraj Institutional Review Board (SIRB), Faculty of Medicine Siriraj Hospital, Mahidol University, Bangkok, Thailand (Certificate of Approval, COA no. Si 826/2022). Informed consent was obtained from all subjects involved in the study. This research followed Consolidated Standards of CONSORT statement and Declaration of Helsinki.

### Genotyping and quality control

Microarray datasets were obtained from the ArrayExpress (E-MTAB-15536). Genotyping was performed using the Axiom Precision Medicine Diversity Array Kit with the Axiom 2.0 Assay (PDMRAv2) (Life Technologies, Foster City, CA, USA) targeting 678 SNPs from ten known T2D-related genes: *CDKAL1*, *CDKN2A*, *CDKN2B*, *HHEX*, * KCNQ1*,*MTNR1B*, *PAX4*, *SLC30A8*, *TCF7L2*, and *UBE2E2*. This genotyping method yielded an average call rate of 99.934% for passing samples and a filtered call rate of 99.88%. After Hardy-Weinberg Equilibrium (HWE) filtering, 376 SNPs remained for analysis.

### Statistical analysis

All statistical analyses were performed using IBM SPSS Statistics version 29.0 (SPSS Inc., Chicago, IL, USA). Clinical parameters: One-way ANOVA and Kruskal-Wallis test. SNP frequencies were compared among clusters using Chi-square or Fisher’s exact tests. Hardy–Weinberg Equilibrium (HWE) was analysed by using Michael H. Court (2005–2008). Multinomial regression analysis was used to examine associations between SNPs and cluster assignments, adjusting for age, with the MARD cluster used as the reference group. A significant *p*-value was less than 0.05.

## Results

### Clinical characteristics

Significant differences among clusters were observed in age, BMI, HbA1c, eGFR, and treatment regimens (Table 1). Table 1 presents the clinical profiles of 471 Thai patients with type 2 diabetes (T2D), classified into four distinct clusters based on established clinical and biochemical parameters: Cluster 1 – Severe Insulin-Deficient Diabetes (SIDD, *n* = 89), Cluster 2 – Metabolic Syndrome-related Diabetes (MSD, *n* = 53), Cluster 3 – Mild Obesity-related Diabetes (MOD, *n* = 118), and Cluster 4 – Mild Age-Related Diabetes (MARD, *n* = 211). There were significant differences in age and age at diagnosis across clusters (*p* < 0.001). Patients in the MARD cluster were the oldest (median age 61.47 years), reflecting age-related onset, while those in the MOD cluster were the youngest (median age 43.5 years), consistent with early-onset obesity-driven diabetes. Sex distribution also differed significantly (*p* = 0.045), with higher proportions of males in the SIDD and MSD clusters, and a lower proportion in MOD. Body mass index (BMI) was highest in the MOD cluster (median 31.69 kg/m²), consistent with its obesity-related profile, and lowest in the SIDD cluster (24.77 kg/m²). Weight and height followed a similar pattern.

**Table 1:** Clinical characteristics in type 2 diabetes clusters.

Variables	All (*n* = 471)	Cluster 1: SIDD (*n* = 89)	Cluster 2: MSD (*n* = 53)	Cluster 3: MOD (*n* = 118)	Cluster 4: MARD (*n* = 211)	*p*-value
Age (years)	54.02 (46.29, 61.78)	53.20 (48.62, 57.80)	50.41 (42.65, 58.83)	43.50 (36.83, 47.39)	61.47 (55.67, 65.04)	**< 0.001** ^**d**^
Age at diagnosis (years)	53 (45, 61)	52 (48, 57)	50 (42, 58)	42 (36, 46)	60 (55, 64)	**< 0.001** ^**d**^
Male (%)	40.3	49.4	47.2	31.4	39.8	**0.045** ^**a**^
SBP (mmHg)	130.70 ± 14.65	127.39 ± 16.69	131.70 ± 14.68	131.55 ± 13.50	131.38 ± 14.24	0.131^c^
DBP (mmHg)	76.04 ± 11.30	74.39 ± 11.83	76.68 ± 10.46	80.19 ± 11.18	74.27 ± 10.78	**< 0.001** ^**c**^
Pulse rate (beats per minute, or bpm)	85.80 ± 13.51	88.43 ± 12.33	88.81 ± 14.43 *n* = 52	89.62 ± 13.40 *n* = 116	81.82 ± 12.81 *n* = 206	**< 0.001** ^**c**^
Height (cm)	160.0 (155.0, 167.0)	160.0 (156.0, 167.5)	163.0 (157.5, 169.5)	161.0 (156.5, 168.0) *n* = 117	160.0 (154.0, 165.0)	**0.017** ^**d**^
Weight (kg)	69.5 (61.2, 80.8)	64.2 (58.3, 70.0)	75.0 (59.9, 84.9)	84.0 (74.8, 92.0)	65.5 (59.9, 73.5)	**< 0.001** ^**d**^
BMI (kg/m^2^)	26.73 (23.76, 30.94)	24.77 (22.44, 26.51)	27.05 (23.72, 31.00)	31.69 (28.69, 35.69) *n* = 117	25.88 (23.28, 29.19)	**< 0.001** ^**d**^
FPG (mg/dL)	152.8 (121.0, 167.0)	144.0 (124.5, 222.8) *n* = 88	141.5 (125.5, 176.0) *n* = 50	142.0 (126.0, 180.5) *n* = 117	130.0 (115.8, 145.5) *n* = 210	**< 0.001** ^**d**^
HbA1c (%)	7.1 (6.5, 8.7)	10.0 (7.4, 12.1) *n* = 86	7.3 (6.6, 10.7) *n* = 50	7.3 (6.6, 8.7) *n* = 115	6.6 (6.3, 7.2) *n* = 207	**< 0.001** ^**d**^
Cholesterol (mg/dL)	183.0 (157.5, 216.0)	186.0 (161.0, 220.0) *n* = 79	191.0 (158.0, 229.0) *n* = 51	189.0 (148.0, 215.5) *n* = 113	180.5 (156.8, 209.0) *n* = 202	0.629^d^
Triglyceride (mg/dL)	140.0 (104.0, 180.0)	124.0 (93.0, 152.0) *n* = 79	286.5 (232.8, 363.3) *n* = 52	144.0 (111.0, 176.0) *n* = 113	126.0 (95.0, 163.5) *n* = 204	**< 0.001** ^**d**^
Calculated LDL (mg/dL)	100.0 (78.0, 132.0)	106.0 (82.0, 142.0) *n* = 71	89.0 (53.8, 135.0) *n* = 38	111.0 (80.5, 133.5) *n* = 101	96.0 (77.5, 121.0) *n* = 189	**0.016** ^**d**^
Direct LDL (mg/dL)	119.2 ± 32.5	115.6 ± 33.0 *n* = 11	117.9 ± 29.4 *n* = 14	115.9 ± 42.7 *n* = 11	124.7 ± 29.5 *n* = 18	
Creatinine (mg/dL)	0.77 (0.62, 0.91)	0.76 (0.59, 0.94) *n* = 84	0.77 (0.65, 0.99) *n* = 48	0.69 (0.59, 0.84) *n* = 102	0.80 (0.66, 0.94) *n* = 201	**< 0.001** ^**d**^
eGFR (ml/min/1.73m^2^)	96.0 (83.0, 106.0)	100.5 (87.3, 109.0) *n* = 84	99.0 (84.0, 111.0) *n* = 47	108.5 (98.3, 115.0) *n* = 100	87.5 (75.0, 97.0) *n* = 200	**< 0.001** ^**d**^
Underlying disease (%)	85.6	77.5	90.6	83.1	89.1	**0.037** ^**a**^
Hypertension disease (%)	53.9	39.3	50.9	53.4	61.1	**0.007** ^**a**^
Dyslipidemia (%)	73.9	65.2	86.8	64.4	79.6	**< 0.001** ^**a**^
Coronary artery disease (%)	2.3	2.2	0	3.4	2.4	0.701^b^
History of diabetes in family (%)	65.8	69.7	71.7	69.5	60.7	0.204^a^
Treatment (%)						
Diet control	16.8	0	9.4	11.9	28.4	**< 0.001** ^**b**^
Sulfonylurea	30.4	60.7	34.0	33.1	15.2	**< 0.001** ^**a**^
Glinide	0.2	0	0	0.8	0	0.552^b^
Metformin	77.1	93.3	79.2	85.6	64.9	**< 0.001** ^**a**^
Thiazolidinedione	4.2	7.9	5.7	5.1	1.9	0.067^b^
DPP4 inhibitor	4.2	4.5	3.8	4.2	4.3	1.00^b^
Alpha glucosidase inhibitor	0	0	0	0	0	-
SGLT2 inhibitor	1.3	1.1	3.8	0.8	0.9	0.417^b^
GLP1 analogue	0	0	0	0	0	-
Insulin	7.0	22.5	7.5	5.1	1.4	**< 0.001** ^**b**^

severe insulin-deficiency diabetes: SIDD; metabolic syndrome diabetes: MSD; mild obesity-related diabetes: MOD; mild age-related diabetes: MARD. p-value were examined by ^a^Chi-Square, ^b^Fisher-Exact Test, ^c^One-way ANOVA and ^d^Kruskal Wallis test. Significant *p*-value was shown in text bold (*p*-value < 0.05).

Glycemic control markers varied markedly. The SIDD cluster had the highest HbA1c (median 10.0%), indicating poor glycemic control and insulin deficiency, whereas the MARD cluster had the lowest HbA1c (median 6.6%, *p* < 0.001). Fasting plasma glucose (FPG) followed the same trend. Blood pressure and lipid parameters also showed significant differences. Although systolic blood pressure (SBP) did not differ significantly (*p* = 0.131), diastolic blood pressure (DBP) and pulse rate were highest in the MOD and MSD clusters (*p* < 0.001), potentially reflecting increased sympathetic tone or metabolic burden. Triglycerides were dramatically elevated in the MSD group (median 286.5 mg/dL), consistent with metabolic syndrome, while other clusters had more modest levels. LDL cholesterol was comparable across clusters, though direct LDL showed variation due to smaller subgroup sizes. Renal function, assessed via creatinine and estimated glomerular filtration rate (eGFR), revealed that the MOD cluster had the highest eGFR (median 108.5 mL/min/1.73 m²), while the MARD cluster had the lowest (median 87.5 mL/min/1.73 m²), aligning with age-related decline.

The prevalence of comorbidities also differed. The MARD cluster had the highest rate of hypertension (61.1%) and dyslipidemia (79.6%), while the SIDD cluster had the lowest hypertension rate (39.3%). Family history of diabetes was common across all groups (~ 66%) without a significant difference. Treatment patterns varied significantly by cluster, reflecting disease severity and phenotype. SIDD patients were most likely to receive insulin (22.5%) and sulfonylureas (60.7%), while MOD and MSD were commonly treated with metformin (> 79%). The MARD group had the highest proportion of patients managed with diet alone (28.4%), suggesting milder disease. Taken together, these findings support the validity of T2D clustering in a Thai population, revealing distinct clinical and therapeutic patterns that align with cluster pathophysiology. These differences provided a foundation for exploring cluster-specific genetic associations in subsequent analyses.

### SNP frequency and cluster association

Out of 376 SNPs that passed quality control, 19 showed statistically significant differences among clusters (Table 2). Among these, eight SNPs demonstrated significant associations with specific T2D clusters after age adjustment in multinomial regression, with the MARD cluster used as the reference group (Table 3). Strongly associated with the MOD cluster was found in patients carrying the GG genotype of *TCF7L2* (rs61875103) (coefficients = 2.34, odds ratio = 10.383). Patients in the SIDD cluster were associated with the AA and AG genotype of rs2283220 (odds ratio = 0.196 and 0.207, respectively) and the CC genotype of rs2074197 (odds ratio = 0.265). Together with the MSD cluster, patients were associated with a heterozygous genotype of rs12576156 (odds ratio = 0.067). Furthermore, the heterozygous genotype of rs2283220 and the homozygous genotype of rs2074197 were correlated with the MOD cluster (odds ratio = 0.159 and 0.208, respectively). Patients with the TC genotype of rs163165 in *KCNQ1* had an increased risk of MSD cluster (coefficient = 3.321 and odds ratio = 27.687). *CDKAL1* (rs4710943, rs9368248, and rs6456379) was associated with reduced odds ratios of being in the SIDD vs. MARD cluster in homozygous wildtype and heterozygous genotype (e.g., rs4710943: CC genotype, odds ratio = 0.208; CT genotype, odds ratio = 0.275).

**Table 2:** Genotyping frequency of genetic polymorphisms in each type 2 diabetes clusters.

Gene chr: position (SNP)	Cluster 1: SIDD			Cluster 2: MSD			Cluster 3: MOD			Cluster 4: MARD			*p*-value
	A/A	A/B	B/B	A/A	A/B	B/B	A/A	A/B	B/B	A/A	A/B	B/B	
*TCF7L2*													
chr10:112961704 (rs61875103)	85	4	0	48	5	0	116	1	0	204	7	0	0.046
chr10:112989975 (rs35011184)	83	5	1	43	10	0	114	4	0	192	19	0	0.006
chr10:112994312 (rs34872471)	80	8	1	41	12	0	111	7	0	187	23	0	0.014
chr10:112996282	80	8	1	41	12	0	110	8	0	188	23	0	0.024
chr10:112998590	80	8	1	41	12	0	110	8	0	189	22	0	0.023
*KCNQ1*													
chr11:2477588 (rs12576156)	66	21	2	42	7	4	88	27	3	145	63	2	0.033
chr11:2589946 (rs112763498)	88	1		51	2		113	5		210	1		0.04
chr11:2609249	85	4	0	49	3	1	105	13	0	200	7	0	0.022
chr11:2614134 (rs75061665)	83	6	0	49	3	1	102	16	0	200	9	1	0.026
chr11:2734318 (rs2283220)	51	30	8	38	13	2	77	30	11	133	73	5	0.033
chr11:2749492 (rs58039093)	73	13	3	40	11	2	91	27	0	169	42	0	0.036
chr11:2804049 (rs2074197)	40	42	6	30	21	2	63	47	7	158	45	8	< 0.001
chr11:2804724 (rs163165)	0	1	88	0	3	50	0	1	117	0	1	210	0.045
*CDKAL1*													
chr6:20739788 (rs4710943)	31	39	19	28	21	4	58	48	12	102	96	13	0.01
chr6:20877272 (rs9368248)	30	37	22	26	22	5	48	56	13	94	102	15	0.002
chr6:20893016 (rs6456379)	28	36	25	23	25	4	45	59	13	85	107	18	0.002
chr6:21003029 (rs9350312)	3	28	58	6	24	23	12	38	68	10	90	111	0.03
chr6:21079478 (rs7750461)	84	4	0	44	9	0	110	8	0	186	25	0	0.044
chr6:21202272 (rs74415845)	80	8	1	41	12	0	104	12	2	174	37	0	0.032

*p*-value was analyzed in T2D clusters by Chi-square test (*n* ≥ 5) or Fisher–Freeman–Halton Exact Test (*n* < 5). Our study selected SNPs only significant differences between T2D clusters.

A/A: Homozygous wildtype; A/B: Heterozygous; B/B: Homozygous mutant.

**Table 3:** Association between SNPs genotype and T2D clusters.

Gene (SNP)	Cluster1: SIDD vs. Cluster4: MARD		Cluster2: MSD vs. Cluster4: MARD		Cluster3: MOD vs. Cluster4: MARD	
	Coefficient	Odds ratio	Coefficient	Odds ratio	Coefficient	Odds ratio
*TCF7L2 gene*						
rs61875103 (G > A)						
GG	0.524	1.688	− 0.067	0.936	**2.34**	10.383*
GA	ref	1.00	ref	1.00	ref	1.00
AA	–	–	–	–	–	–
*KCNQ1* *gene*						
rs12576156 (A > G)						
AA	− 0.742	0.476	− 1.9	0.15	− 0.872	0.418
AG	− 0.924	0.397	**− 2.703**	0.067*	− 1.022	0.36
GG	ref	1.00	ref	1.00	ref	1.00
rs2283220 (A > G)						
AA	**− 1.631**	0.196*	− 0.483	0.617	− 1.363	0.256
AG	**− 1.575**	0.207*	− 0.993	0.37	**− 1.839**	0.159*
GG	ref	1.00	ref	1.00	ref	1.00
rs2074197 (C > T)						
CC	**− 1.328**	0.265*	− 0.654	0.52	**− 1.568**	0.208*
CT	− 0.104	0.901	0.172	1.187	− 0.602	0.548
TT	ref	1.00	ref	1.00	ref	1.00
rs163165 (T > C)						
TT	–	–	–	–	–	–
TC	1.273	3.572	**3.321**	27.687*	1.54	4.667
CC	ref	1.00	ref	1.00	ref	1.00
*CDKAL1* *gene*						
rs4710943 (C > T)						
CC	**− 1.569**	0.208**	− 0.012	0.988	− 0.317	0.728
CT	**− 1.292**	0.275*	− 0.317	0.728	− 0.665	0.514
TT	ref	1.00	ref	1.00	ref	1.00
rs9368248 (A > C)						
AA	**− 1.626**	0.197**	− 0.231	0.794	− 0.53	0.589
AC	**− 1.546**	0.213**	− 0.605	0.546	− 0.742	0.476
CC	ref	1.00	ref	1.00	ref	1.00
rs6456379 (C > A)						
CC	**− 1.531**	0.216**	0.205	1.227	− 0.224	0.799
CA	**− 1.415**	0.243**	0.126	1.134	− 0.208	0.812
AA	ref	1.00	ref	1.00	ref	1.00

*p-value < 0.05, **pvalue < 0.001; Multinomial regression analysis was adjusted with age.

### Allele-level associations

Analysis at the allele level confirmed key findings, as shown in Table 4. For allele-specific of *TCF7L2* (rs61875103), the major allele (G allele) was significantly associated with MOD cluster (coefficient = 2.290, odds ratio = 9.877). The C allele of rs2074197 in *KCNQ1* was protective in SIDD, MSD, and MOD clusters (odds ratio = 0.377, 0.523, and 0.404, respectively). For rs163165 (T > C) in *KCNQ1*, patients with the T allele in the MSD cluster had significantly higher frequencies, 26.063 times, compared to the T allele in the MARD cluster. Moreover, the major allele of rs4710943, rs9368248, and rs6456379 in *the CDKAL1* gene were associated with a protective SIDD cluster (odds ratio = 0.539, 0.538, and 0.525, respectively).

**Table 4:** Allele frequency for risk variants in T2D clusters.

Gene (SNP)	Cluster1: SIDD vs. Cluster4: MARD		Cluster2: MSD vs. Cluster4: MARD		Cluster3: MOD vs. Cluster4: MARD	
	Coefficient	Odds ratio	Coefficient	Odds ratio	Coefficient	Odds ratio
*TCF7L2 gene*						
rs61875103 (G > A)						
G allele	0.505	1.657	-0.064	0.938	**2.290**	9.877*
A allele	ref	1.00	ref	1.00	ref	1.00
*KCNQ1* *gene*						
rs12576156 (A > G)						
A allele	0.044	1.045	0.017	1.017	0.016	1.016
G allele	ref	1.00	ref	1.00	ref	1.00
rs2283220 (A > G)						
A allele	− 0.362	0.696	0.271	1.312	− 0.036	0.965
G allele	ref	1.00	ref	1.00	ref	1.00
rs2074197 (C > T)						
C allele	**− 0.977**	0.377**	**− 0.648**	0.523*	**− 0.905**	0.404*
T allele	ref	1.00	ref	1.00	ref	1.00
rs163165 (T > C)						
T allele	1.260	3.526	**3.261**	26.063*	1.497	4.468
C allele	ref	1.00	ref	1.00	ref	1.00
*CDKAL1* *gene*						
rs4710943 (C > T)						
C allele	**− 0.618**	0.539*	0.145	1.156	0.066	1.068
T allele	ref	1.00	ref	1.00	ref	1.00
rs9368248 (A > C)						
A allele	**− 0.620**	0.538*	0.086	1.089	− 0.068	0.934
C allele	ref	1.00	ref	1.00	ref	1.00
rs6456379 (C > A)						
C allele	**− 0.645**	0.525*	0.099	1.104	− 0.041	0.960
A allele	ref	1.00	ref	1.00	ref	1.00

*p-value < 0.05, **pvalue < 0.001; Multinomial regression analysis was adjusted with age.

## Discussion

This study highlights key genetic variants that may underpin the heterogeneity in T2D pathogenesis. *TCF7L2* is consistently associated with beta-cell dysfunction, aligning with its enrichment in the MOD cluster. *KCNQ1* and *CDKAL1* variants—linked to insulin secretion and resistance—were differentially distributed across clusters. These findings support a model in which specific genetic risk profiles may inform cluster membership, with implications for personalised treatment strategies.

This study is among the first in Thailand to explore the genetic architecture of clinically defined T2D clusters using high-throughput SNP genotyping. By analysing variants in 10 well-established T2D susceptibility genes^6^, we identified eight SNPs—primarily in *TCF7L2*,* KCNQ1*, and *CDKAL1*—that were significantly associated with specific T2D clusters. These findings reinforce the hypothesis that genetically distinct pathways contribute to the pathophysiological differences among T2D subtypes.

Our results align with and extend earlier cluster-based diabetes studies, such as those by Ahlqvist et al. (2018), who first proposed the clustering model in a Swedish cohort^14^. While the original study focused on clinical progression and complication risk, subsequent efforts attempted to link T2D clusters to genetic risk scores derived from genome-wide association studies (GWAS)^15,16^. These studies consistently showed that certain clusters, particularly SIDD and MOD, exhibit stronger associations with beta-cell function and obesity-related loci, respectively. However, most of these investigations were conducted in European populations, limiting their generalizability to Southeast Asian cohorts.

In our Thai population, we found that *TCF7L2* rs61875103, a well-known beta-cell function variant, was strongly associated with the MOD cluster, suggesting that even among individuals with obesity-driven T2D, beta-cell dysfunction contributes substantially. This contrasts slightly with findings from European cohorts, where *TCF7L2* variants were more often linked with insulin-deficient forms (e.g., SIDD)^16^. This discrepancy may reflect population-specific gene-environment interactions or differences in the age of onset and BMI distribution in Asian populations.

Variants in *KCNQ1*, a gene implicated in insulin secretion, showed robust associations with both SIDD and MSD clusters in our study. For example, rs2074197 (C > T) and rs2283220 (A > G) were protective in SIDD and MOD, suggesting a shared mechanistic role involving impaired beta-cell signalling and insulin resistance. Previous studies in East Asian populations have consistently highlighted *KCNQ1* as a major contributor to T2D susceptibility^17,18^, often with larger effect sizes compared to those seen in Europeans. The enrichment of *KCNQ1* variants in our insulin-deficient and insulin-resistant subtypes supports these earlier findings and highlights the gene’s multifaceted role.

*CDKAL1*, another beta-cell gene, was also strongly associated with the SIDD cluster in our study. SNPs such as rs4710943 and rs9368248 were significantly more frequent in SIDD compared to MARD, even after adjusting for age. These results are consistent with data from both GWAS and functional studies, indicating that *CDKAL1* variants reduce insulin synthesis and secretion, particularly in Asian populations where early-onset diabetes with low BMI is more prevalent^19^. Interestingly, our study showed no strong *CDKAL1* association with the MOD cluster, underscoring the specificity of this gene’s role in insulin-deficient diabetes. It is also notable that clinical characteristics differed sharply among clusters, consistent with previous reports. SIDD patients in our cohort had the highest HbA1c and lowest BMI, while MOD individuals had the highest BMI and eGFR. MARD patients, the oldest group, had the mildest glycemic and lipid profiles. Treatment patterns also followed expected trends, with insulin use concentrated in the SIDD group^20^, while diet-only management was more common in MARD. These phenotypic differences strengthen the biological relevance of clustering and support its use as a framework for precision medicine.

Compared to other studies, our use of the Axiom PDMRAv2 array—designed for metabolic and pharmacogenomic research—allowed for high-resolution genotyping in genes with both common and rare variants. The application of Hardy-Weinberg filtering and age-adjusted multinomial regression enhanced the robustness of our associations. Despite the strengths, this study has limitations. First, its cross-sectional design precludes assessment of longitudinal outcomes or treatment response by genotype. Second, while we focused on 10 known T2D genes, whole-genome or exome sequencing could reveal additional loci relevant to T2D clusters in Southeast Asians. Third, functional validation of identified SNPs remains necessary to confirm causal mechanisms.

**Fig. 1:**
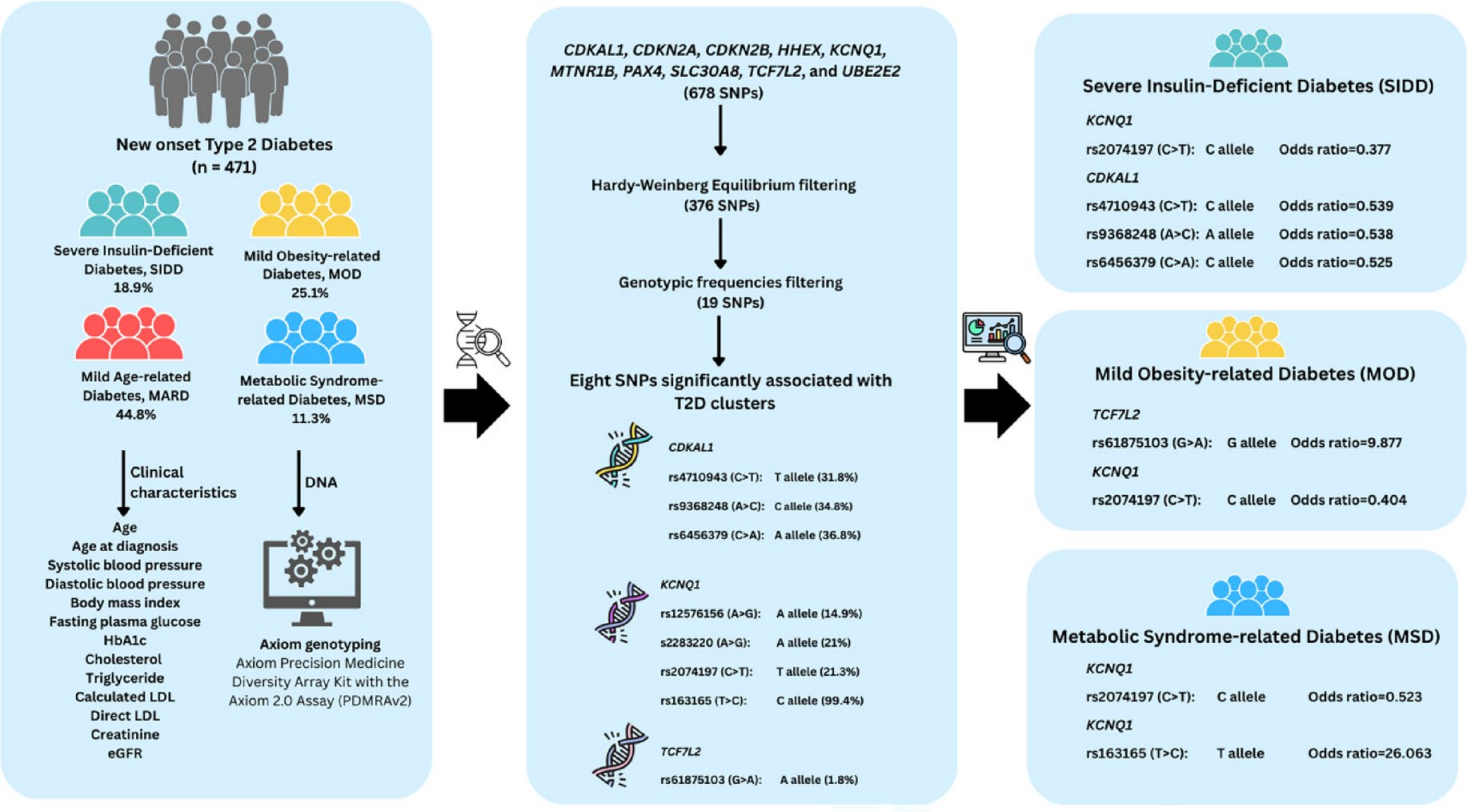
Genetic association of SNPs with type 2 diabetes (T2D) clusters. Among 471 newly diagnosed T2D patients classified into Severe Insulin-Deficient Diabetes (SIDD, 18.9%), Mild Obesity-related Diabetes (MOD, 25.1%), Mild Age-related Diabetes (MARD, 44.8%, reference), and Metabolic Syndrome-related Diabetes (MSD, 11.3%), DNA samples were genotyped using the Axiom Precision Medicine Diversity Array Kit (PDMRAv2). From 678 SNPs across 10 T2D-associated genes, eight variants were significantly linked to clusters. Protective SNPs in *KCNQ1* and *CDKAL1* were associated with SIDD, while *TCF7L2* rs61875103 (G allele, OR = 9.877) and *KCNQ1* rs2074197 (C allele, OR = 0.404) were linked to MOD. For MSD, *KCNQ1* rs2074197 and rs163165 (T allele, OR = 26.063) showed significant associations, supporting genetic heterogeneity in T2D subgroups.

In conclusion, our findings provide evidence of distinct genetic associations with clinically meaningful T2D clusters in the Thai population. The enrichment of *TCF7L2*, *KCNQ1*, and *CDKAL1* variants in specific subtypes highlights the potential for integrating genetic screening into diabetes stratification models. In the future, all variants of *TCF7L2*,* KCNQ1*, and *CDKAL1* could be genotyped to classify clusters of newly diagnosed type 2 diabetes (T2D) in large, multi-centre studies with long-term follow-up. This approach could ultimately inform individualised treatment strategies and improve disease management outcomes in diverse populations (Fig. 1).

## Conclusion

The identification of cluster-specific genetic associations underscores the relevance of integrating genotyping data into T2D management. These insights pave the way for precision medicine approaches tailored to genetic profiles within clinical T2D subtypes.

## Data Availability

The raw genotyping microarray datasets are available in ArrayExpress under accession number E-MTAB-15536. All data analyzed in this study are included within this published article.
